# Dataset of PAHs determined in home-made honey samples collected in Central Italy by means of DLLME-GC-MS and cluster analysis for studying the source apportionment

**DOI:** 10.1016/j.dib.2022.108136

**Published:** 2022-04-06

**Authors:** Sergio Passarella, Ettore Guerriero, Luisangela Quici, Giuseppe Ianiri, Marina Cerasa, Ivan Notardonato, Carmela Protano, Matteo Vitali, Mario Vincenzo Russo, Antonio De Cristofaro, Pasquale Avino

**Affiliations:** a Department of Agricultural, Environmental and Food Sciences (DiAAA), University of Molise, via De Sanctis, I-86100 Campobasso, Italy; b Institute of Atmospheric Pollution Research (IIA), National Research Council (CNR), Rome Research Area-Montelibretti, I-00015 Monterotondo Scalo, Italy; c Department of Agricultural and Forest Sciences (DAFNE), University of Tuscia, Via San Camillo de Lellis, I-01100 Viterbo, Italy; d Department of Public Health and Infectious Diseases, University of Rome “La Sapienza”, p.le Aldo Moro 5, I-00185 Rome, Italy

**Keywords:** Honey, PAH, Bioindicator, DLLME, GC-MS, Cluster analysis, PCA, Source apportionment

## Abstract

This paper would like to show all the data related to an intensive field campaign focused on the characterization of the Polyaromatic Hydrocarbons (PAHs) composition profile in almost 60 honey samples collected in Central Italy. The analytical data here reported are the base for a study aimed to identify the pollution sources in a region. 22 PAHs were analyzed by means of ultrasound-vortex-assisted dispersive liquid-liquid micro-extraction (DLLME) procedure followed by a triple quadrupole gas chromatograph/mass spectrometer (GC-MS). A chemometrics approach has been carried out for evaluating all the data: in particular, principal component analysis and cluster analysis has been used both for the identification of the main natural/anthropogenic pollutants affecting a site and for evaluating the air quality.

## Specifications Table

**Table:** 

Subject	Food Science: Food Chemistry Analytical Chemistry Environmental Chemistry Pollution
Specific subject area	Chromatography, food quality, air quality monitoring, source apportionment
Type of data	Tables Figures Chromatograms
How the data were acquired	The data were acquired by a triple quadrupole gas chromatograph/mass spectrometer Other data from scientific literature
Data format	Raw Analyzed Filtered
Description of data collection	The honey samples were processed by an extraction protocol based on the ultrasound-vortex-assisted dispersive liquid-liquid micro-extraction (DLLME) procedure followed by a gas chromatography coupled with a triple quadrupole mass spectrometry. The analyses were carried out using a standard solution of perdeuterated PAH compounds.
Data source location	All the samples were collected in Central Italy, regions Latium and Molise; the analyses were carried out at the laboratories of the Institute of Atmospheric Pollution Research (IIA), National Research Council (CNR), in Rome. Belgrade and Serbia database was available in literature.
Data accessibility	The dataset is available on this article and can be found in Mendeley repository data: Passarella, Sergio; Guerriero, Ettore; Quici, Luisangela; Ianiri, Giuseppe; Cerasa, Marina; Notardonato, Ivan; Protano, Carmela; Vitali, Matteo; Russo, Mario V.; De Cristofaro, Antonio; Avino, Pasquale (2022), “Raw chromatogram files of honey samples analyzed by GC-MS/QqQ”, Mendeley Data, V1, doi: 10.17632/kn72vrxxy7.1 https://data.mendeley.com/datasets/kn72vrxxy7/1
Related research article	**For an article which has been submitted:** S. Passarella, E. Guerriero, L. Quici, G. Ianiri, M. Cerasa, I. Notardonato, C. Protano, M. Vitali, M.V. Russo, A. De Cristofaro, P. Avino, PAH source apportionment in home-made honey samples collected in Central Italy by means of chemometric approach, Food Chem. 382 (2022) 132361. doi: 10.1016/j.foodchem.2022.132361 https://www.sciencedirect.com/science/article/abs/pii/S0308814622003235?via%3Dihub

## Value of the Data

• The analytical procedure reported allows to investigate PAHs by perdeuterated compounds and DLLME-GC-MS analysis at trace levels
• Honey samples can be considered as a biomonitoring index in anthropogenic or natural areas, avoiding long and tedious sampling procedures
• Data can be useful for source apportionment of PAHs in relationship to different emissions for air quality studies
• Data can used by other scientists for different chemometrics analysis in the food quality study

## 1 Data Description

The dataset reported here is related to the analytical procedure set up for analyzing 22 polyaromatic hydrocarbons (PAHs) (Table 1) in honey samples adapted from Kazazic et al. [1].

**Table 1:** List of the PAHs investigated and the related acronyms.

PAH	Acronym
Acenaphthene	Acy
Acenaphthylene	Ace
Anthracene	Ant
Benzo[a]anthracene	BaA
Benzo[a]pyrene	Bb+jF
Benzo[b+j]fluoranthene	BkF
Benzo[e]pyrene	BghiP
Benzo[ghi]perylene	BaP
Benzo[k]fluoranthene	BeP
Chrysene	Chr
Dibenzo[a,e]pyrene	DahA
Dibenzo[a,h]anthracene	DalP
Dibenzo[a,h]pyrene	DaeP
Dibenzo[a,i]pyrene	DaiP
Dibenzo[a,l]pyrene	DahP
Fluoranthene	Fu
Fluorene	Fl
Indeno[1,2,3-cd]pyrene	IPy
Naphthalene	Na
Perylene	Phe
Phenanthrene	Py
Pyrene	Per

The raw files of the gas chromatography coupled with mass spectrometry (GC-MS) data are available in a dedicated repository: all the chromatograms are deposited in the Mendeley one [2]. It should be noted that in the repository 62 chromatograms are deposited: the difference, i.e. 5 chromatograms, is due to samples #2997 and #2998 whose chromatographic runs were repeated three times, and to a toluene chromatogram reported (for checking the column clearness).

Under such analytical conditions 57 home-made honey samples were analyzed. For a preliminary analysis of the relations among PAHs, the Pearson's correlation was performed: Table 2 shows the main correlations between PAHs with R above 0.6.

**Table 2:** Main correlations between PAHs, showing an R above 0.6. For acronyms: see Table 1. Table adapted from ref. [3].

Correlations between 0.6-0.7	Correlations between 0.7-0.8	Correlations between 0.8-0.9	Correlations > 0.9
Ace-Acy	Fl-Acy	BeP-BkF	B(b+j)F-Chr
Ant-Phe	Fl-Ace	BaP-Chr	IPy-B(b+j)F
Py-Fu		BaP-B(b+j)F	BghiP-B(b+j)F
BaA-Phe		DahA-BaA	BghiP-IPy
B(b+j)F-Fu		IPy-Chr	DaIP-Per
BaP-BkF		IPy-BaP	DaeP-Per
Per-BaP		BghiP-Chr	DaeP-DaIP
DahA-Phe		BghiP-BaP	DaiP-Per
DahA-Fu			DaiP-DalP
IPy-BkF			DaiP-DaeP
DaeP-DahA			DahP-Per
			DahP-DalP
			DahP-DaeP
			DahP-DaiP

The simultaneous presence of 22 PAHs and 57 samples generates a problem of multivariate analysis. Before running the chemometric approach, the analysis of variance (ANOVA) was carried out by SPSS statistics software for Windows, version 25.0 (IBM Corp., Armonk, NY, USA). The results show what compounds with high concentration variability (i.e., high relative standard deviation, RSD) record high square mean values, and a significance value (or α level) equal to zero (< 0.01), i.e. BaA, BeP, Bb+jF, BghiP, BkF, IPy, Chr, BaP, DahA, Phe, DalP, DaiP, DahP, DaeP, Per. The main consideration regards the role of molecules at the highest molecular weight. In fact, this occurrence is responsible for the sample distribution in different clusters. The Cluster Analysis (CA), performed by means of SPSS software and based on non-hierarchical (k-means) technique, meaning that the grouping is built on Euclidean distance, was applied for determining the possible grouping among the honey samples [4]. First, 4 clusters were identified. Table 3 shows distance among center clusters: the greater the distance between the final centers of the clusters, the greater their dissimilarity.

**Table 3:** Distance among center clusters.

Cluster	1	2	3	4
1		60.21	63.54	58.04
2	60.21		27.53	27.60
3	63.54	27.53		27.04
4	58.04	27.60	27.04	

On the other hand, Table 4 reports the number of samples in each cluster. It can be noted that cluster 1 is characterized by 1 sample (#41), cluster 2 by 3 samples (#32, #49, #50) and cluster 4 by 6 samples (#15, #19, #24, #25, #47, #53) whereas the cluster 3 is the most abundant containing 41 samples.

**Table 4:** Number of samples (#) in each cluster.

Cluster	# Sample	Quote %
1	1	2.0
2	3	5.9
3	41	80.4
4	6	11.8
*valid*	*51*	*100.0*

Table 5, Table 6, Table 7 show the statistical data (in terms of mean, min, max values, standard deviation, RSD and 95 percentile) of each cluster (except for cluster 1).

**Table 5:** Minimum, maximum and mean value (expressed as ng g^−1^) along with sd, RSD% and 95 percentile (ng g^−1^) of each PAH in cluster 2.

	Cluster 2						
PAH	# sample	Min	Max	Mean	sd	RSD%	95 percentile
Acenaphthene	3	0.021	0.17	0.10	0.08	75.7	0.17
Acenaphthylene	3	0.056	0.20	0.13	0.07	55.9	0.19
Anthracene	3	0.071	0.14	0.10	0.04	37.6	0.14
Benzo[a]anthracene	3	0.023	0.63	0.40	0.33	82.3	0.62
Benzo[a]pyrene	3	0.000	0.57	0.35	0.30	87.9	0.56
Benzo[b+j]fluoranthene	3	0.000	0.35	0.21	0.18	88.3	0.34
Benzo[e]pyrene	3	0.457	3.15	2.22	1.52	68.8	3.14
Benzo[ghi]perylene	3	0.006	0.51	0.18	0.29	161.6	0.46
Benzo[k]fluoranthene	3	0.787	1.82	1.38	0.53	38.5	1.79
Chrysene	3	0.000	0.03	0.02	0.02	91.6	0.03
Dibenzo[a,e]pyrene	3	0.009	0.73	0.25	0.42	165.4	0.66
Dibenzo[a,h]anthracene	3	0.007	0.55	0.19	0.31	165.6	0.49
Dibenzo[a,h]pyrene	3	0.001	1.00	0.34	0.57	171.5	0.90
Dibenzo[a,i]pyrene	3	0.001	1.00	0.34	0.57	171.5	0.90
Dibenzo[a,l]pyrene	3	0.000	1.00	0.34	0.57	170.6	0.90
Fluoranthene	3	0.188	0.27	0.22	0.04	19.6	0.26
Fluorene	3	0.034	0.35	0.19	0.16	84.2	0.34
Indeno[1,2,3-cd]pyrene	3	0.003	0.52	0.33	0.28	86.1	0.51
Naphthalene	3	0.026	0.69	0.30	0.35	116.4	0.64
Perylene	3	0.000	0.64	0.21	0.36	169.6	0.57
Phenanthrene	3	1.081	1.41	1.21	0.17	14.1	1.38
Pyrene	3	0.246	0.36	0.29	0.06	20.8	0.35

**Table 6:** Minimum, maximum and mean value (expressed as ng g^−1^) along with sd, RSD% and 95 percentile (ng g^−1^) of each PAH in cluster 3.

	Cluster 3						
PAH	# sample	Min	Max	Mean	sd	RSD%	95 percentile
Acenaphthene	41	0.00	1.70	0.40	0.39	96.9	1.21
Acenaphthylene	41	0.03	2.84	0.71	0.68	95.2	1.81
Anthracene	41	0.02	0.14	0.08	0.03	37.9	0.13
Benzo[a]anthracene	41	0.00	0.86	0.12	0.19	159.4	0.52
Benzo[a]pyrene	41	0.00	0.25	0.04	0.05	130.3	0.12
Benzo[b+j]fluoranthene	41	0.00	0.58	0.13	0.11	87.4	0.31
Benzo[e]pyrene	41	0.01	0.62	0.11	0.14	127.8	0.45
Benzo[ghi]perylene	41	0.00	0.42	0.07	0.10	140.6	0.26
Benzo[k]fluoranthene	41	0.00	0.90	0.07	0.14	210.2	0.18
Chrysene	41	0.01	0.46	0.09	0.09	98.0	0.25
Dibenzo[a,e]pyrene	41	0.00	0.07	0.01	0.01	178.4	0.04
Dibenzo[a,h]anthracene	41	0.00	0.22	0.02	0.04	209.7	0.09
Dibenzo[a,h]pyrene	41	0.00	0.04	0.00	0.01	171.3	0.01
Dibenzo[a,i]pyrene	41	0.00	0.04	0.00	0.01	171.3	0.01
Dibenzo[a,l]pyrene	41	0.00	0.09	0.01	0.01	212.6	0.01
Fluoranthene	41	0.12	0.44	0.22	0.06	27.8	0.30
Fluorene	41	0.03	2.06	0.65	0.51	78.8	1.47
Indeno[1,2,3-cd]pyrene	41	0.00	0.31	0.05	0.07	137.1	0.18
Naphthalene	41	0.00	1.89	0.26	0.32	122.8	0.64
Perylene	41	0.00	0.03	0.01	0.01	131.2	0.03
Phenanthrene	41	0.70	1.49	1.09	0.18	16.4	1.46
Pyrene	41	0.19	0.86	0.45	0.19	41.6	0.78

**Table 7:** Minimum, maximum and mean value (expressed as ng g^−1^) along with sd, RSD% and 95 percentile (ng g^−1^) of each PAH in cluster 4.

	Cluster 4						
PAH	# sample	Min	Max	Mean	sd	RSD%	95 percentile
Acenaphthene	6	0.013	0.507	0.297	0.195	65.6	0.496
Acenaphthylene	6	0.030	2.272	0.826	0.757	91.6	1.903
Anthracene	6	0.028	0.111	0.064	0.034	52.1	0.109
Benzo[a]anthracene	6	0.069	0.975	0.592	0.402	67.9	0.953
Benzo[a]pyrene	6	0.410	1.130	0.743	0.264	35.5	1.072
Benzo[b+j]fluoranthene	6	1.017	1.691	1.371	0.256	18.6	1.680
Benzo[e]pyrene	6	0.584	1.193	0.884	0.204	23.0	1.147
Benzo[ghi]perylene	6	0.833	1.963	1.361	0.428	31.4	1.874
Benzo[k]fluoranthene	6	0.638	1.134	0.863	0.166	19.2	1.081
Chrysene	6	0.615	1.387	1.079	0.281	26.0	1.372
Dibenzo[a,e]pyrene	6	0.074	0.180	0.130	0.046	35.3	0.178
Dibenzo[a,h]anthracene	6	0.082	0.197	0.138	0.039	27.8	0.188
Dibenzo[a,i]pyrene	6	0.029	0.118	0.054	0.037	68.1	0.109
Dibenzo[a,h]pyrene	6	0.029	0.118	0.054	0.037	68.1	0.109
Dibenzo[a,l]pyrene	6	0.032	0.118	0.069	0.033	48.5	0.110
Indeno[1,2,3-cd]pyrene	6	0.660	1.434	1.039	0.292	28.1	1.386
Fluoranthene	6	0.183	0.524	0.334	0.115	34.5	0.488
Fluorene	6	0.024	0.731	0.428	0.251	58.6	0.696
Naphthalene	6	0.007	1.565	0.420	0.625	148.6	1.358
Perylene	6	0.026	0.220	0.127	0.084	66.3	0.217
Phenanthrene	6	0.856	1.386	1.095	0.175	15.9	1.326
Pyrene	6	0.392	1.179	0.626	0.298	47.6	1.069

A Principal Component Analysis (PCA) was applied for identifying the similarities among different datasets [5,6]. The chemometrics approach was carried out by open-access software, i.e. Tanagra [7]: the only condition considered was to have a dataset made of the same compounds. Following this statement, 15 PAHs were considered for the chemometrics treatment. The authors performed the PCA overall three datasets. In details, Table 8 shows the PCA applied to all the samples (i.e., 135 samples, divided in 51 from this study, in 61 from Serbia area [8] and in 23 from Belgrade area [9]).

**Table 8:** PCA of all the samples investigated in this study along with the data collected in other papers [8,9].

	Eigenvalue			Extraction Sums of Squared Loadings		
Component	Total	Variance %	Cumulative %	Total	Variance %	Cumulative %
1	8.865	59.100	59.100	8.865	59.100	59.100
2	2.288	15.254	74.354	2.288	15.254	74.354
3	1.425	9.501	83.855	1.425	9.501	83.855
4	0.920	6.131	89.986			
5	0.835	5.566	95.552			
6	0.372	2.481	98.033			
7	0.121	0.808	98.840			
8	0.113	0.755	99.596			
9	0.030	0.199	99.794			
10	0.023	0.153	99.948			
11	0.005	0.037	99.984			
12	0.001	0.009	99.993			
13	0.001	0.004	99.997			
14	0.000	0.002	99.999			
15	0.000	0.001	100.000			

**Fig. 1:**
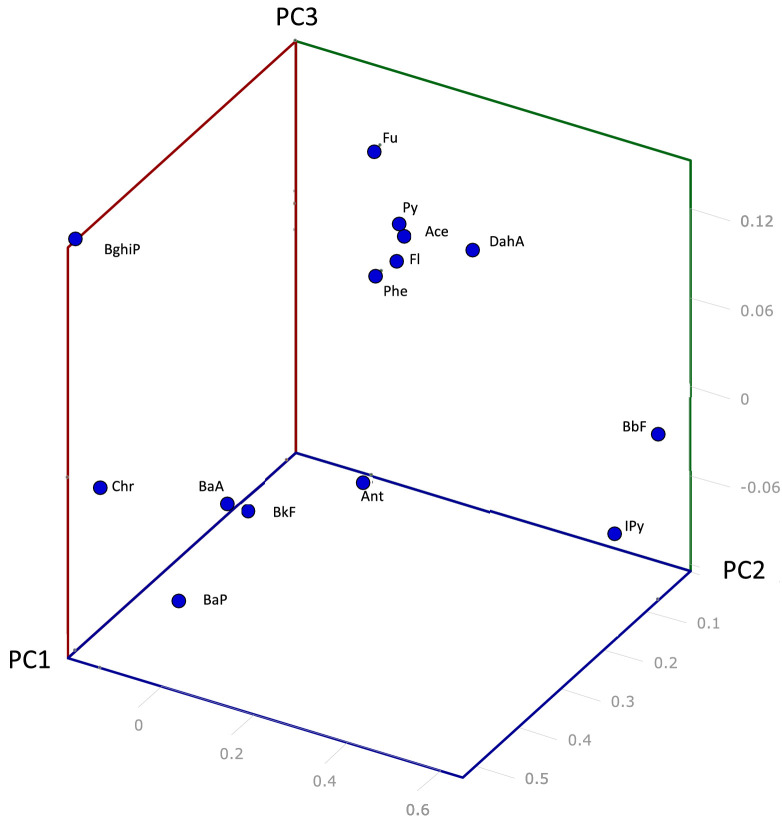
3-D PCA-plot for each PAH using all the data. For acronyms: see Table 1. Figure modified from ref. [3].

**Fig. 2:**
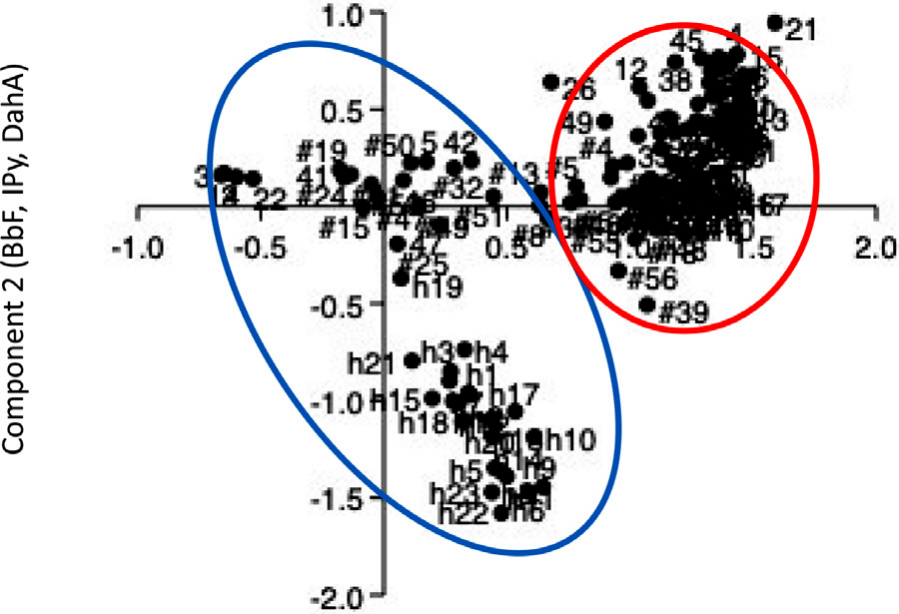
PCA score plot of the samples collected both in Belgrade and in Serbia and in Central Italy (this study) (“sample”: data from Serbia area [8]; #”sample”: this study; h“sample”: data Belgrade area [9]). For acronyms: see Table 1. Figure modified from ref. [3].

Fig. 1, obtained applying the PCA to all the common PAHs, shows the 3-D PCA-plot for identifying the different relevant contribution of each one whereas the Fig. 2 shows the PCA biplot applied to all the samples investigated in the three studies, using the two principal components.

## 2 Experimental Design, Materials and Methods

### 2.1 Honey sample collection

**Fig. 3:**
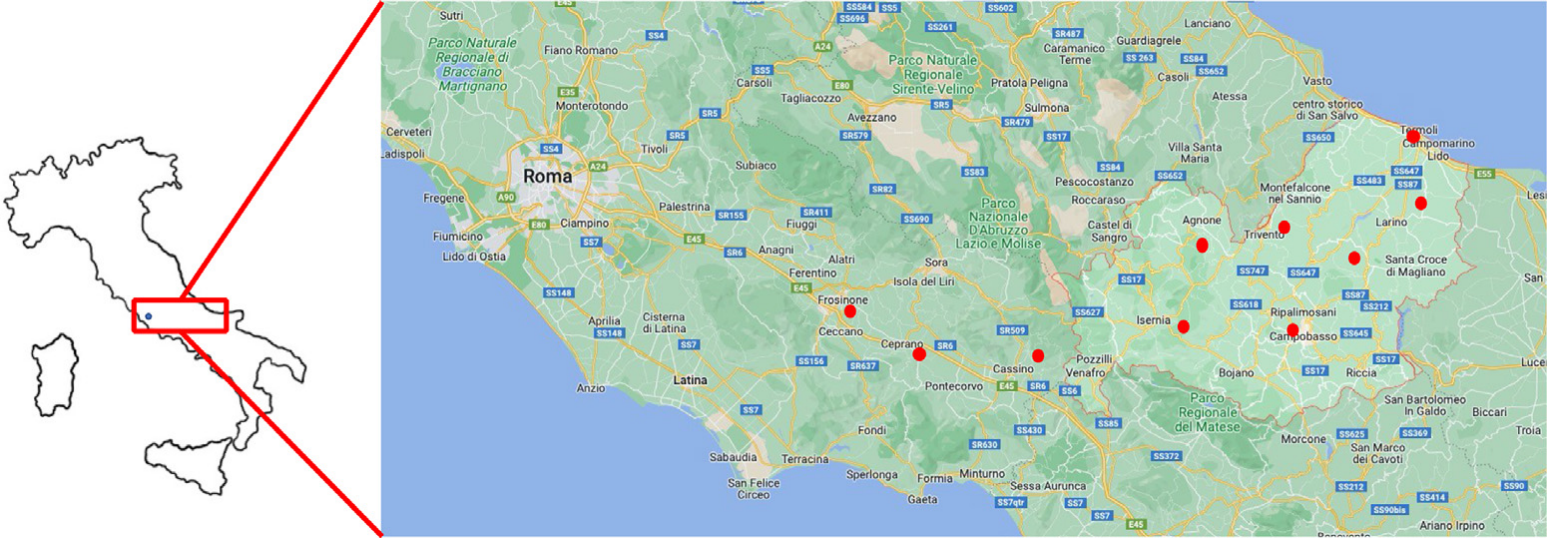
In the map the sampling sites were reported (red circles).

The analysis involved 57 home-made honey samples from different geographical locations in Central Italy. The sampling was carried out in maritime, hilly and mountainous areas; the samples were directly collected in the apiaries by local experts in the period from May to July. The samples were collected in different locations reported in Fig. 3; in each sampling site 5 samples were withdrawn every 15 days. For a better understanding of the PAH behavior in terms of distribution and contamination, a comparison with other data present in literature was carried out. In particular, two papers were considered: they report a dataset of PAHs determined in honey samples collected in Serbia [8] and Belgrade [9] areas. These are the only papers showing a complete PAH profile.

### 2.2 DLLME procedure

The extraction was carried out by DLLME procedure [10]. 10 µL of the extraction standard solution of perdeuterated PAHs (10 ng µL^−1^, L 429 IS, Wellington Laboratories) were added to 2.5 g of honey sample in acetone solution before shaking by vortexing for 40 seconds to favor the dissolution of the sample. The microextraction was performed using 150 μL of chloroform and the extraction process was favored by the formation first of a macroemulsion by vortexing for 5 min and then by the formation of a microemulsion with the aid of an ultrasonic bath for 6 min. Subsequently, in order to facilitate the breaking of the emulsion and the recovery of the solvent, 10 g L^−1^ of NaCl were added and then centrifuged at 4000 rpm for 30 minutes [11]: after, 1 µL was injected.

### 2.3 PAHs analysis by GC-MS

The instrumental analyses were performed by a triple quadrupole gas chromatograph/mass spectrometer (GC-MS) (Trace 1310 GC/TSQ 8000 Evo) (Thermo Fisher Scientific, Waltham, MA, USA) in electronic impact (EI) mode and the chromatographic separation was performed by a DB-XLB column (60 m  ×  0.25 mm, 0.25 μm I.D.) (Agilent Technologies, Santa Clara, CA, USA) with H_2_ 3.00 mL min^−1^ as the carrier gas. The PTV splitless injector was maintained at a constant temperature of 250 °C, mass transfer line temperature of 290°C and ion source temperature of 300°C. The oven was held at 60 °C for 1 min, then warmed 20 °C min^−1^ until 200°C was reached, and held for 0 min, after was warmed at 7.0°C min^−1^ until 275°C and held for 7 min, finally it was warmed at 18 C min^−1^ until 325°C and held for 13 min. The analysis was performed in Selected Ion monitoring (SIM) and full scan mode: SIM time 0.215 s, full scan mode time 0.083 s and total scan mode time 0.300 s.

**Fig. 4:**
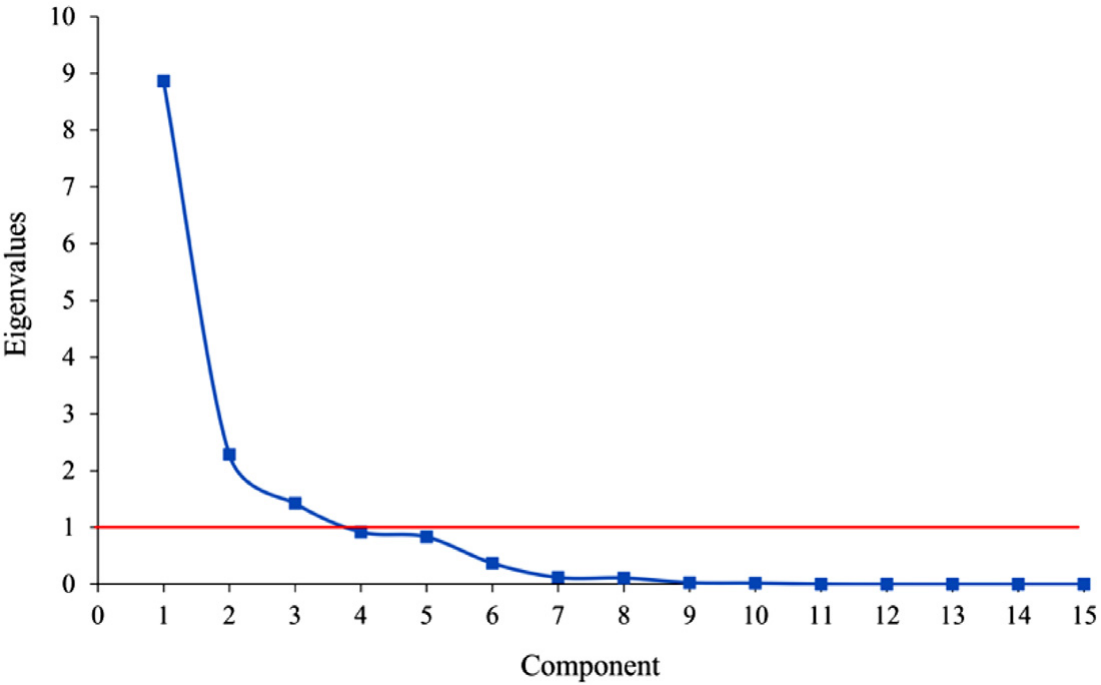
Scree graph of the entire dataset, reporting the eigenvalue.

The first three components (Table 8), chosen because the eigenvalue is above 1 (Fig. 4) [12], describe 84 % of the whole dataset.

## CRediT Author Statement

Pasquale Avino: Conceptualization; Giuseppe Ianiri, Ivan Notardonato: Investigation; Ettore Guerriero, Marina Cerasa: Formal analysis; Luisangela Quici, Sergio Passerella: Software; Mario Vincenzo Russo: Validation; Carmela Protano, Matteo Vitali: Data Curation; Pasquale Avino: Writing - Reviewing & Editing; Antonio De Cristofaro: Supervision*.*

## Funding

This research was supported by the grant BEEOBSERVER “Biodiversità e biomonitoraggio ambientale”.

## Declaration of Competing Interest

The authors declare that they have no known competing financial interests or personal relationships that could have appeared to influence the work reported in this paper.

## Data Availability

Raw chromatogram files of honey samples analyzed by GC-MS/QqQ (Original data) (Mendeley Data)
